# Possible cytokine biomarkers in pediatric acute appendicitis

**DOI:** 10.1186/s13052-019-0726-7

**Published:** 2019-10-15

**Authors:** Nikola Stankovic, Maja Surbatovic, Ivan Stanojevic, Radoje Simić, Slavisa Djuricic, Maja Milickovic, Blagoje Grujic, Djordje Savic, Vesna Milojkovic Marinovic, Miona Stankovic, Danilo Vojvodic

**Affiliations:** 1 Mother And Child Health Care Institute of Serbia, Radoja Dakica 6, Belgrade, 11000 Serbia; 2 Faculty of Medicine, University of Belgrade, Belgrade, Serbia; 3 Military Medical Academy, Belgrade, Serbia; 4 Faculty of Medicine of the Military Medical Academy, University of Defence, Belgrade, Serbia; 5 Banjaluka University School of Medicine, Banjaluka, Bosnia and Herzegovina; 6 Novartis, Serbia

**Keywords:** Biomarker, Cytokine, Acute appendicitis, Children

## Abstract

**Background:**

Diagnosis of acute appendicitis (AA) and decisions about its treatment remain among the most common dilemmas of pediatric surgical teams. Monitoring of immune response may be of importance for this purpose. Our aim was to measure and analyze serum and peritoneal fluid cytokines, in children who had undergone surgery for suspected AA.

**Methods:**

Prospective investigation of serum and peritoneal fluid cytokine values was performed in 127 consecutive patients. According to the pathohistological findings, patients were divided into three groups: normal/early, uncomplicated and complicated AA. Determination of cytokine concentrations for 20 different cytokines was done using a commercial flow cytometry kit: Human Inflammation 20 plex BMS 819.

**Results:**

Statistically significant differences in serum cytokine values between pathohistological groups were found for IP-10, MIP-1α and IL-10. Preoperative cut-off values of IP-10, MIP-1α and IL-10 between groups were obtained using ROC curve analysis. Positive correlations between serum and peritoneal concentrations were recorded for most of the analyzed cytokines.

**Conclusion:**

IP-10, MIP-1α and IL-10 showed potential in assessment of AA in children. Confirmatory studies with a larger number of patients are required to prove reliability of these biomarkers.

## Introduction

There is no clear guidance to drive a clinical decision between conservative and surgical treatment of acute appendicitis (AA) in children. This results in significant number of unnecessary surgeries [1] or appendiceal perforations, classifying AA as one of the most frequently misdiagnosed conditions in pediatric surgery. Contributing factors include poor clinical history, insufficient cooperation during physical examination, and the medical team’s fear of perforative appendicitis risk [2].

Introduction of imaging methods has improved diagnostics, but without the desired reduction in negative appendectomy rate [1, 3] and complicated forms of appendicitis [4]. Ultrasonography usually gives insufficient information, while computerized tomography is linked to high doses of ionizing radiation, and its use is therefore questionable in terms of benefit versus risk.

Standard laboratory parameters such as white blood count (WBC) with leukocyte formula and C-reactive protein (CRP) are not specific and sensitive enough for diagnosis of AA [5]. New laboratory technologies and the development of immunology improve monitoring of inflammatory processes, and allow searching for specific biomarkers in order to optimize diagnosis of AA [6–8].

Inflammation in AA includes activation of immune cells, and their complex interaction is mediated by a number of cytokines. Cytokines are predominantly secreted by macrophage cells and T-lymphocytes, and this large group of proteins, peptides, or glycoproteins can be classified as pro-inflammatory or anti-inflammatory according to their function in immune response.

So far, investigations involving measurement of cytokines in AA have been conducted predominantly in adults [9–11], with only a small number in pediatric patients [12, 13].

In this prospective trial, we analyzed serum and peritoneal cytokine concentrations in children referred for surgery due to suspected AA, with the aim of identifying mediators that could improve diagnosis of AA in children.

## Patients and methods

During the period April–December 2015, patients admitted to the Mother and Child Healthcare Institute of Serbia with a clinical diagnosis of AA requiring open surgery were eligible for prospective evaluation within this trial. The following population was not eligible: children under 3 years or older than 16 years; patients with other acute diseases; patients with an operative finding of other abdominal inflammation; and patients referred for laparoscopic surgery. Informed consent from a parent or legal guardian was obtained for all patients included in this trial. The trial was approved by an Institutional Ethics Committee, and run in line with Good Clinical Practice and the Declaration of Helsinki.

A total of 127 patients were stratified into three groups, according to pathohistological findings. The first group comprised patients with a normal appendix or early stage appendicitis (NEAA), where a normal appendix was found. The second group comprised patients with phlegmonous or uncomplicated appendicitis (UAA). The third group comprised patients with gangrenous and/or perforated appendicitis, classed as complicated appendicitis (CAA).

Baseline evaluation was done on the day of surgery, and included blood and peritoneal fluid sampling. Serum obtained by peripheral venous blood centrifugation was taken before surgery, and supernatant obtained by centrifugation of peritoneal fluid was taken just after laparotomy; both were stored at -70C for later measurement of cytokines. Two additional blood samplings were performed at the 1st and the 3rd postoperative day for the same laboratory analysis. All resection samples obtained were sent for histological processing.

Determination of the cytokine concentrations in the sera and peritoneal fluid supernatants was performed on a Beckman Coulter FC500 cytometer, according to the manufacturer’s instructions of the commercial flow cytometry kit: Human Inflammation 20 plex BMS 819. It contains reactants for determination of the following cytokines: sE-selectin, G-CSF, ICAM-1, IFN-α, IFN-γ, IL-1α, IL-1β, IL-4, IL-6, IL-8, IL-10, IL-12p70, IL-13, IL-17A, IP-10, MCP-1, MIP-1α, MIP-1β, LAP and TNF-α.

PRISM GraphPad software version 5.01 was used for statistical analysis. Correlations (Spearman rho) and comparisons (Mann–Whitney U-test) were calculated for comparative statistics (z-score and two-tailed P).

The study was approved by the Ethics Committee of the Mother and Child Healthcare Institute of Serbia, and run in line with Good Clinical Practice and the Declaration of Helsinki.

## Results

There were 77 male and 50 female patients from 3 to 16 years old (on average 10.43 ± 4.02), distributed as shown in Table 1.

**Table 1:** Demographic characteristic*s* and distribution within groups (NEAA – normal or early acute appendicitis; UAA – uncomplicated acute appendicitis; CAA – complicated acute appendicitis)

	3–8 years old		9–12 years old		13–16 years old		total	
	boys	girls	boys	girls	boys	girls	boys	girls
NEAA (*n* = 20)	5	3	2	2	2	6	9	11
UAA (*n* = 30)	4	6	8	1	6	5	18	12
CAA (*n* = 77)	22	8	12	9	16	10	50	27
total	31	17	22	12	24	21	77	50
	48		34		45		127	

The accuracy of surgeons’ intraoperative diagnosis (percentage of pathohistologicaly confirmed intraoperative findings) was 73.2%, while the incidence of negative appendectomy (absence of inflammation in the appendix after surgical intervention for suspected appendicitis) was 15.7%.

### Preoperative differences of serum cytokine values between pathohistological groups

**Fig. 1:**
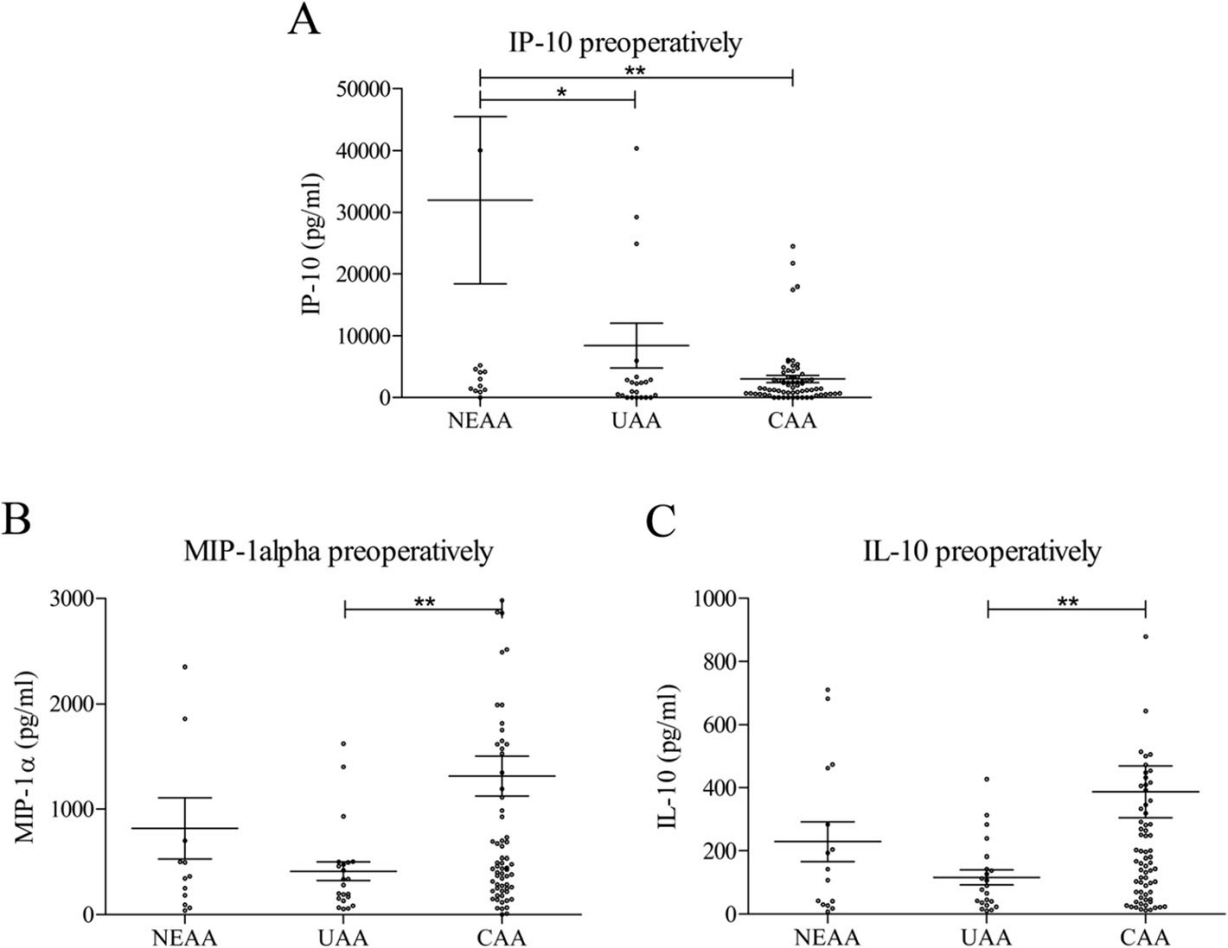
Preoperative comparison of serum cytokine values between pathohistological groups: IP-10 (**a**); MIP-1α (**b**); IL-10 (**c**). NEAA – normal or early acute appendicitis; UAA – uncomplicated acute appendicitis; CAA – complicated acute appendicitis. [mean ± standard error of mean (SEM), Mann-Withney test, *p < 0.05, **p < 0.01, ***p < 0.001]

Statistically significant differences between preoperative serum cytokine values of the pathohistological groups were found for IP-10, MIP-1α and IL-10 (Fig. 1). The other 17 examined cytokines did not show a statistically significant difference preoperatively (not shown).

In the preoperative samples, the highest values of IP-10 were found in NEAA, while the lowest were recorded in CAA group (Fig. 1 A). Values of IP-10 in the NEAA group were significantly higher than values in UAA (31,962 ± 54,209 vs. 8437 ± 17,431, *p* = 0.0410) and CAA group (31,962 ± 54,209 vs. 3099 ± 4771, *p* = 0.0099). Statistical significance was not reached in comparison of the preoperative IP-10 values between the UAA and CAA groups.

MIP-1α had the highest preoperative values in CAA, and the lowest in UAA (Fig. 1 B). Significant statistical difference was recorded only between the UAA and CAA groups (412 ± 414 vs. 1113 ± 1618, *p* = 0.0065).

Preoperative IL-10 values were the highest in CAA, and the lowest in UAA (Fig. 1 C), showing significant statistical difference (116 ± 111 vs. 386 ± 695, *p* = 0.0079), while significant differences were not found in comparisons of these two groups with the NEAA group.

### IP-10, MIP-1α and IL-10 cut-off values between pathohistological groups

**Fig. 2:**
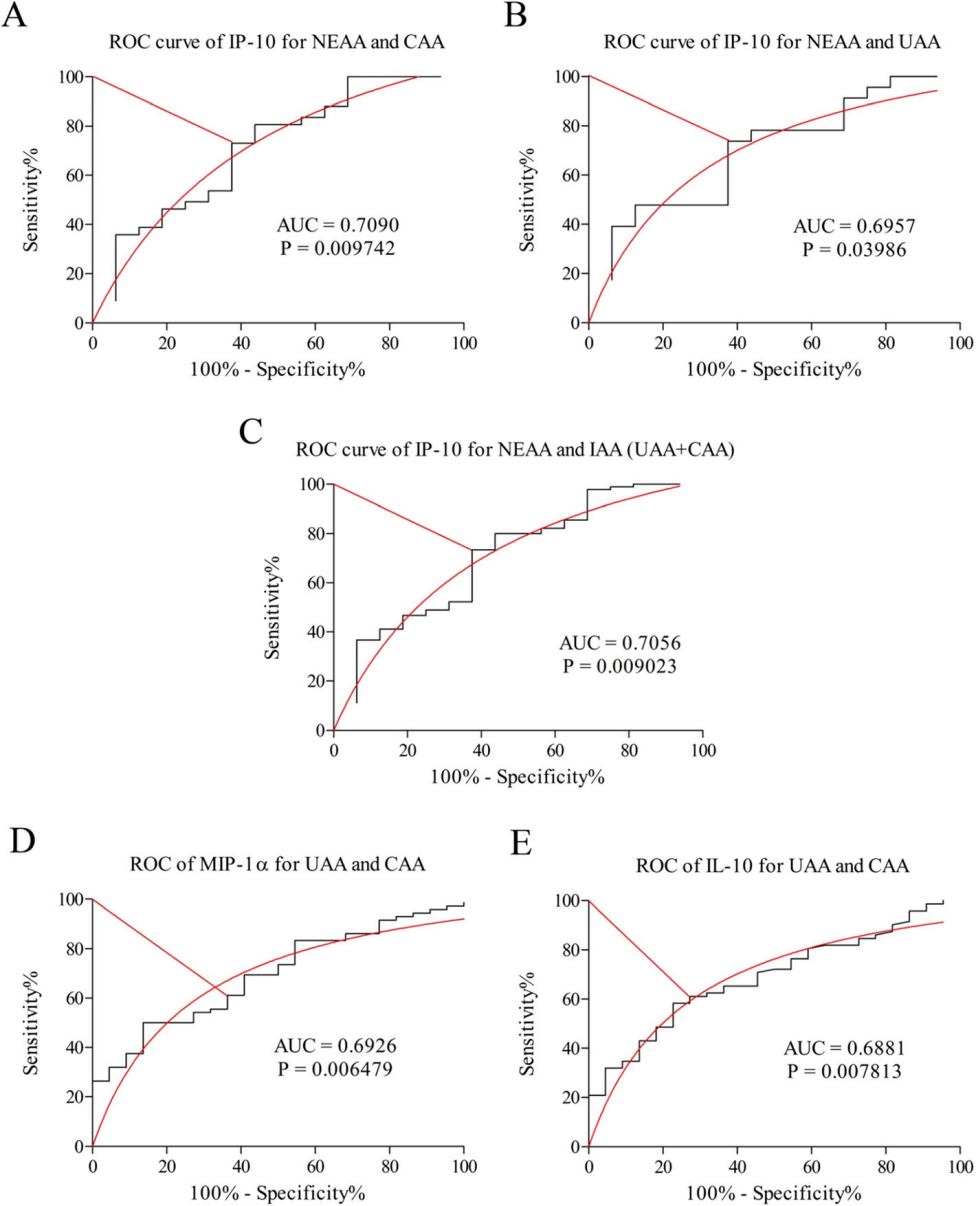
ROC (Receiver Operating Characteristic) curves of cytokines for formed pathohistological groups. NEAA – normal or early acute appendicitis; UAA – uncomplicated acute appendicitis; CAA – complicated acute appendicitis; IAA – inflamed acute appendicitis. ROC curve of IP-10 for NEAA and UAA (**a**); ROC curve IP-10 for NEAA and CAA (**b**); ROC curve of IP-10 for NEAA and IAA (UAA + CAA) (**c**); ROC curve of MIP-1α for UAA and CAA (**d**); ROC curve of IL-10 for UAA and CAA (**e**)

The optimal cut-off value of IP-10 between the NEAA and UAA groups was 2956 pg/ml, with sensitivity of 73.91% and specificity of 62.5% (AUC = 0.7090, *p* = 0.009742; Fig. 2 A). For the NEAA and CAA groups, the optimal IP-10 cutoff value was 2994 pg/ml, with sensitivity of 73.13% and specificity of 62.5% (AUC = 0.6957, *p* = 0.03986; Fig. 2 B). The optimal cutoff value of IP-10 between the NEAA and IAA (inflamed acute appendix; UAA + CAA) groups was also 2994 pg/ml, with sensitivity of 73.53% and specificity of 62.18% (AUC = 0.8175, *p* < 0.0001; Fig. 2 C).

In the case of MIP-1α, the optimal cut-off value between the UAA and CAA groups was 424 pg/ml, with sensitivity of 61.11% and specificity of 63.64% (AUC = 0.6926, *p* = 0.006479; Fig. 2 D).

Optimal cut-off value of IL-10 between the UAA and CAA groups was 130 pg/ml, with sensitivity of 62.50 and specificity of 68.18 pg/ml (AUC = 0.6881, *p* = 0.007813, Fig. 2 E) .

All five conducted tests could be considered as tests with moderate accuracy.

### Differences in serum cytokine values between pathohistological groups on the 1st postoperative day

**Fig. 3:**
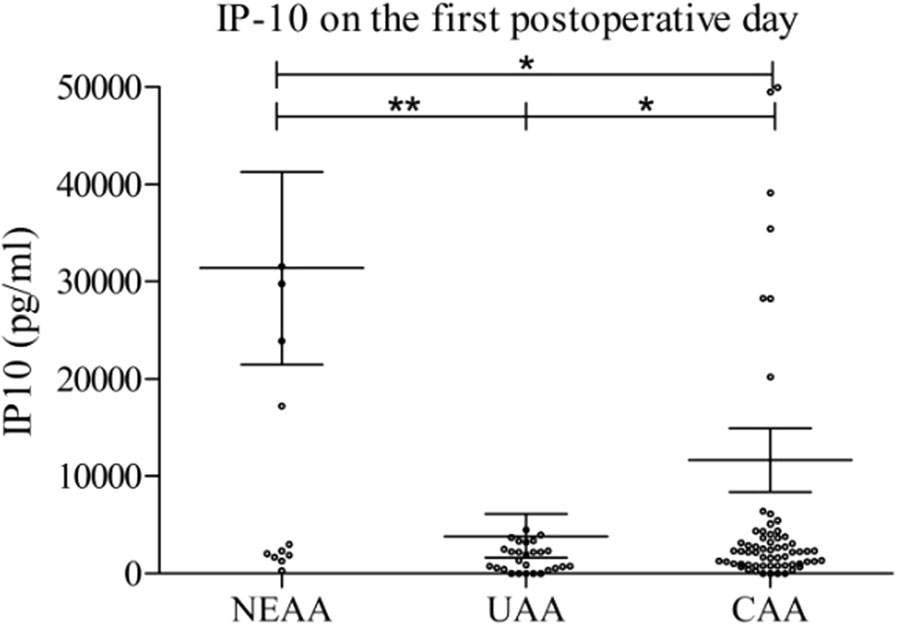
Comparison of IP-10 values between pathohistological groups on the first postoperative day. NEAA – normal or early acute appendicitis; UAA – uncomplicated acute appendicitis; CAA – complicated acute appendicitis. [mean ± standard error of mean (SEM), Mann-Withney test, *p < 0.05, **p < 0.01, ***p < 0.001]

On the first postoperative day, a difference in cytokine values between pathohistological groups was recorded only in the case of IP-10, with the highest values in NEAA and the lowest in UAA (Fig. 3). These values discriminated between the three pathohistological groups. Values in the NEAA group were significantly higher than in UAA (31,404 ± 38,365 vs. 3868 ± 11,810, *p* = 0.0049) or in CAA (31,404 ± 38,365 vs. 11,658 ± 27,013, *p* = 0.0281). Similarly, statistically significant difference was found between UAA and CAA (3868 ± 11,810 vs. 11,658 ± 27,013, *p* = 0.0364). The other 19 examined cytokines did not show a statistically significant difference on the first postoperative day (not shown).

### Differences in serum cytokine values between pathohistological groups on the 3rd postoperative day

**Fig. 4:**
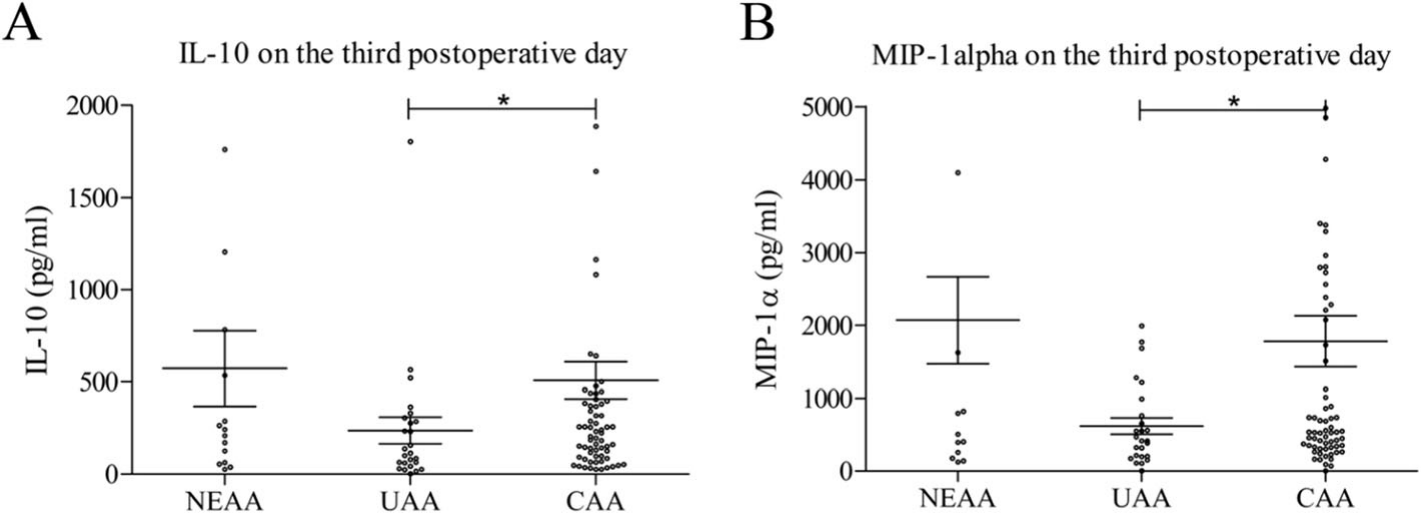
Comparison of cytokine values between pathohistological groups on the third postoperative day: IL-10 (**a**); MIP-1α (**b**). NEAA – normal or early acute appendicitis; UAA – uncomplicated acute appendicitis; CAA – complicated acute appendicitis. [mean ± standard error of mean (SEM), Mann-Withney test, *p < 0.05, **p < 0.01, ***p < 0.001]

On the third postoperative day, significant differences between groups in serum cytokine concentrations were found only for IL-10 and MIP-1α (Fig. 4). The other 18 examined cytokines did not show a statistically significant difference (not shown).

Concentrations of IL-10 were highest in NEAA and lowest in UAA (Fig. 4 A). Values in UAA were significantly lower compared to CAA (236 ± 362 vs. 508 ± 834, *p* = 0.0430), but not compared to NEAA. Statistically significant differences were not found between the NEAA and UAA values.

MIP-1α values showed similar relations between groups as IL-10 values (Fig. 4B). Significant difference was recorded only between UAA and CAA (619 ± 557 vs. 1779 ± 2869, *p* = 0.0352).

### Preoperative correlations of serum and peritoneal cytokine values in the total population

**Fig. 5:**
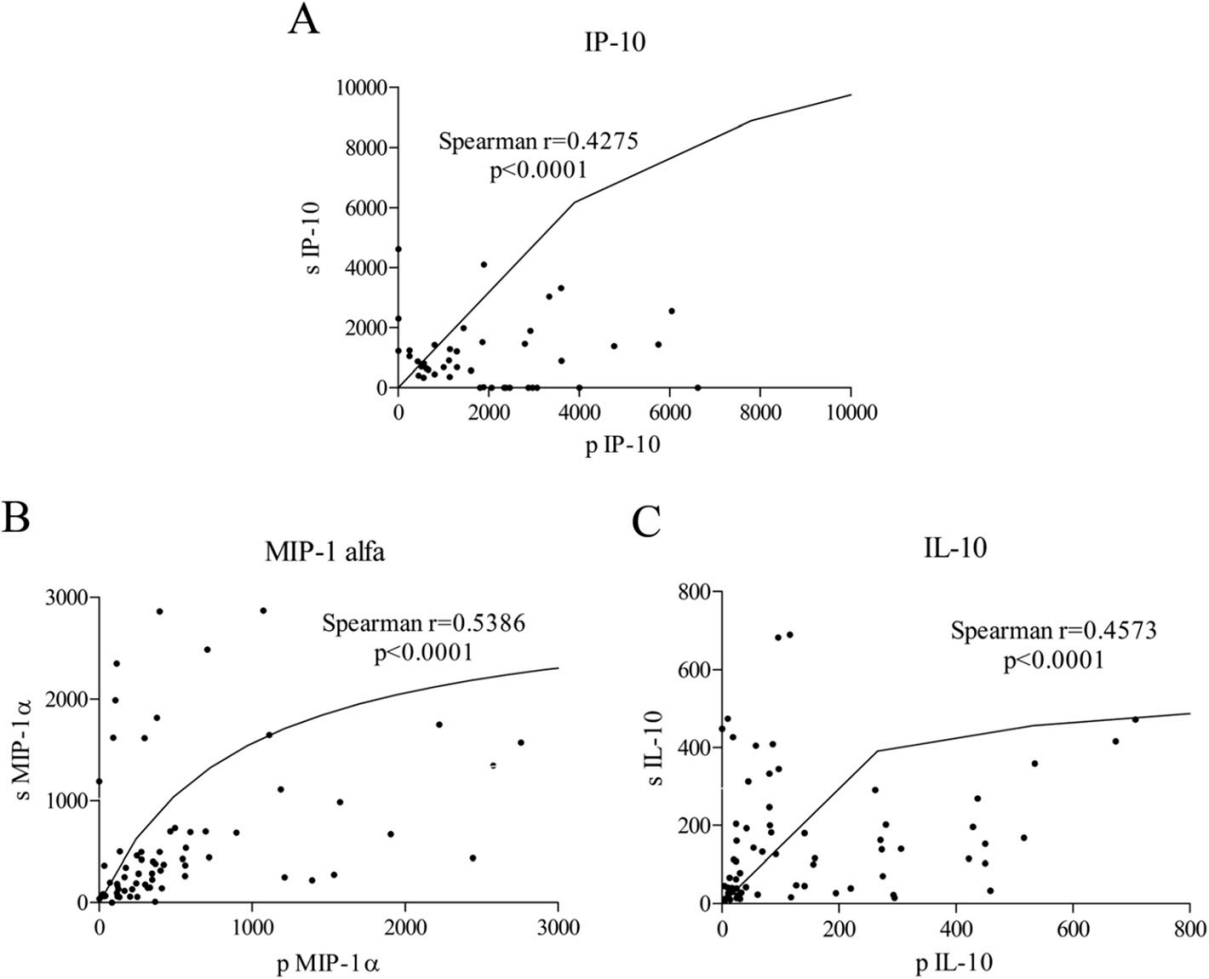
Correlation of preoperative serum and peritoneal cytokine values on total patients: IP-10 (**a**); MIP-1α (**b**); IL-10 (**c**)

Preoperative serum and peritoneal concentrations showed strong positive correlations for IP-10 (Spearman r = 0.4275, *p* < 0.0001, Fig. 5 A), MIP-1α (Spearman r = 0.5386, p < 0.0001, Fig. 5 B) and IL-10 (Spearman r = 0.4573, p < 0.0001, Fig. 5 C). All other investigated mediators in this study, with exception of IL-6, also demonstrated positive correlation between their serum and peritoneal values (not shown).

## Discussion

Reliability in diagnosis of AA is improved by combining clinical and imaging methods with WBC and CRP values, which are the laboratory gold standard. As imaging methods are time consuming and may involve exposure to radiation, and echosonography is usually insufficiently informative [14]. A combination of physical examination and laboratory tests therefore remains the gold standard, although no laboratory parameters show sufficient sensitivity and specificity [5, 15]. For instance, acute mesenteric lymphadenitis has the most similar clinical presentation to AA.

Neutrophils represent one of the first lines of defense against penetrating agents. These phagocytes secrete lytic enzymes and produce free oxygen radicals with high antimicrobial potential. Their activation is triggered by bacteria, and by secreted cytokines and chemokines. The number of neutrophils in the blood grows by mobilization of marginal pool and bone marrow, in proportion to the extent of inflammation. Lymphocytes are immunocompetent cells that coordinate immune response and assist in neutrophil activation. CRP is an acute inflamatory phase reactant synthesized in the liver under control of IL-6. Along with positive physical and radiological findings, it may have good diagnostic value in AA. However, as an isolated parameter it is not useful, because of low specificity [5]. On the other hand, the first increase in CRP occurs 12 h after inflammation starts, with peak plasma concentration between 24 to 48 hours [16]. Some studies suggest that CRP is an important diagnostic agent for perforated AA but not for AA in general [17].

Laboratory parameters which include the value of neutrophils, such as absolute neutrophil count, percentage of neutrophils in the leukocyte formula, and neutrophil-to-lymphocyte ratio (NLR), are considered as a better diagnostic agent in AA, because neutrophils rise faster than CRP which needs time for synthesis in the liver [18]. Some studies showed that NLR appears to have greater diagnostic accuracy than WBC [19]. In our previous publication we confirmed a faster activation of neutrophils and NLR increase, and delayed increase of CRP, in pediatric AA [20, 21]. Therefore usefulness of NLR in early diagnosis of AA is superior to CRP, but still not sufficiently reliable. In the gangrenous form of AA, significant lymphopenia may be described whose pathophysiological mechanism is not fully understood [22, 23]. In accordance with this, the increase in the value of NLR in developed form of AA occurs as a result of the increase in the number of neutrophils as well as in reduction of the number of lymphocytes.

Monitoring of immune response and cytokine profiles may be of importance in the diagnosis of AA. Identifying highly specific biomarkers for certain stages of AA would make clinical decisions much easier. Similarly to our study, other investigations have aimed to identify potential biomarker functions of serum cytokines, in order to determine the presence or absence of the inflammatory process and the degree of inflammation in the appendix [24–26]. The clinical significance of these potential serum biomarkers could include: reducing the number of negative appendectomies, better assessment of treatment, choice of antibiotic therapy and surgical techniques, better planning of recovery, and reduced overall hospital costs. However, based on current knowledge, there is no reliable biomarker with the above characteristics [14]. Even though other studies have explored this problem, none of them used the comprehensive set of 20 different cytokines (determined by Human Inflammation 20 plex BMS 819) for sample testing, as was done within this trial.

Yoon et al. evaluated five cytokine molecules, including pro-inflammatory IL-1β, IL-6 and IL-8, IL-2 and anti-inflammatory IL-10 [27]. The results suggested the importance of IL-6 and IL-8 in the differentiation of perforative and non-perforative acute appendicitis. Although in our patients, concentrations of these cytokines in serum were elevated, especially in the CAA group, the difference was not significant and their differential potential was not confirmed. In another study with mixed age groups of patients, the usefulness of IL-10 for this purpose was presented [8], confirming the conclusions of several previous studies [28–30].

The results of this trial identify 3 out of 20 tested serum cytokines, IL-10, MIP-1α and IP-10, which showed statistically different concentrations between pathohistological groups.

Interleukin 10 (IL-10) is an anti-inflammatory cytokine primarily produced by activated macrophages and Th2-cells. Its role is to reduce antigen-presenting capacity of cells and to inhibit Th1 immune response. At the same time, IL-10 potentiates Th2 response, enhancing the proliferation of B-cells and synthesis of antibodies [31]. According to the findings of this study, IL-10 was the only interleukin whose level varied significantly between UAA and CAA. Pre-operative serum levels of IL-10 were significantly higher in CAA, which can be explained by enhanced Th2 activity in the developed form of AA.

Macrophage inflammatory protein 1α (MIP-1α) belongs to a family of chemokines primarily produced by macrophage cells activated by bacterial endotoxin, and has a crucial role in immune response to infection [32]. The pro-inflammatory role of this cytokine is reflected in the activation of granulocytes, and the induction of synthesis of other pro-inflammatory cytokines. Results of this study show that values of MIP-1α differentiated UAA and CAA in a similar manner as IL-10. This chemokine plays significant role in attraction and activation of granulocytes, and its serum concentration was expectedly higher in advanced AA. These are the first published results of MIP-1α monitoring in AA.

Interferon inducible protein 10 (IP-10) is a chemokine secreted by many cells stimulated by IFN-γ, such as monocytes, fibroblasts and endothelial cells. The biological function of IP-10 is attraction of mononuclear phagocytes and promotion of Th1 immune response [33, 34]. Its activity is directly correlated to Th1 response, and accordingly its serum concentrations may be of value in monitoring of immune processes [35]. Results of this trial demonstrate that serum concentrations of IP-10 could distinguish NEAA from the other two groups, but could not distinguish between UAA and CAA. As expected, when we correlated serum levels of IP-10 with levels of IFN-γ, which is a well-known inducer of IP-10, we found strong positive correlation in the UAA and CAA groups (*p* = 0.0003, Spearman r = 0.6801 and *p* < 0.0001 Spearman r = 0.6500, respectively, not shown). In contrast, in the NEAA patient group, which otherwise showed statistically highest IP-10 values, there was no statistically significant correlation of IP-10 with IFN-γ. In addition, the IFN-γ levels showed no statistically significant differences between the NEAA, UAA and CAA groups. Taken together, these findings indicate the possibility that the prompt upregulation of IP-10 in the NEAA patient group could be achieved by mechanisms other than IFN-γ mediated induction. Interpretations of IP-10 levels in AA have rarely been published.

The triple biomarker set we describe, composed of one anti-inflammatory cytokine and two chemokines, could be useful in clinical assessment of AA in children, as an “add-on” diagnostic test to other standard diagnostic tools for AA. IP-10 assessment can be used in order to confirm exclusion of AA, while IL-10 and MIP-1α can be tools for differentiation between uncomplicated and complicated AA. Cutoff values obtained by ROC curve analysis in this trial were classified as tests with poor to moderate reliability. However, a study with a larger and more uniform patient group could provide better reliability.

The accuracy of surgeons’ intraoperative diagnosis was 73.2% within the setting of this trial, and this is similar to other published results [36]. The largest discrepancy between intraoperative and histological findings was in the case of perforative appendicitis. This is likely due to micro perforations, which are not visible macroscopically during the surgery. These specimens are sent to the pathologist with an intraoperative diagnosis of uncomplicated appendicitis, but micro perforations are detected later by microscope, leading to classification as a complicated form of AA.

The incidence of negative appendectomy was 15.7%, which is significantly lower than previously published results in adult and pediatric patients [37, 38].

Evaluation of cytokine serum concentration after surgery could serve for monitoring of recovery and potential development of complications. According to Eriksson et al., reliable estimation of complications could be based on monitoring of leukocytes, CRP and IL-6 [39]. The only cytokine that separated groups on the first postoperative day in this study was IP-10. On the third postoperative day, concentrations of IL-10 and MIP-1α were able to separate UAA and CAA, but not NEAA from these two groups. Thus, in the three-day postoperative period of the study, the same cytokine variables could distinguish between diagnostic groups. A few studies have evaluated IL-10 after surgery for AA [8, 13, 40, 41], but there is no available data for MIP-1α and IP-10.

In this study, analysis of peritoneal cytokine concentrations was done in peritoneal fluid samples taken at the beginning of surgery, immediately after the peritoneal cavity had been opened. Single peritoneal fluid sampling allowed analysis of group differentiation but not analysis of post-operative cytokine value changes, as had been the case for blood sampling for serum cytokines. Separation of groups on the basis of peritoneal cytokine concentrations is well described. Local processes within AA can have be reflected systemically with variable intensity. It can be completely restricted, and thus without systemic effects and corresponding serum changes, or can be transmitted to a systemic level to a lesser or greater extent. The effects of pro-inflammatory cytokines can be transmitted to a systemic level, increasing the risk of an inflammatory reaction in previously intact organs and tissues [42–44]. Conversely, locally created pro-inflammatory cytokines can induce suppression of systemic inflammation and prevent inflammation in other tissues [45–48].

Although a large number of investigations were conducted to qualify and quantify local and systemic immune response, and to determine the pro- or anti-inflammatory character of response in AA, this study was aimed to evaluate and differentiate stages of AA and potential clinical benefit in diagnosis and follow up of children with AA. Peritoneal cytokine concentrations are more likely than serum concentrations to differentiate pathohistological groups, even if peritoneal samples are impossible to obtain before a surgical procedure.

There is significant positive correlation between serum and peritoneal cytokine values for most of the tested cytokines. However, only 3 out of 20 cytokines from this investigation showed sufficient differences in serum values to differentiate among described groups of patients.

The main limitation of this study is imbalance between pathohistological groups, especially the numerical domination of patients with complicated AA. Cytokine kinetics and the half-life of certain cytokines in plasma, as well as differences between their in vivo and in vitro activities, can be considered as other potential limiting factors.

## Conclusion

The surgeon’s clinical examination remains the most important factor in the diagnosis of AA in children. The possibility of distinguishing preoperatively between different stages of AA in children, based on serum cytokine levels, would be of great value in clinical practice. According to the results of this study, IL-10, MIP-1α and IP-10 showed potential to help achieve this goal. In conjunction with clinical examination, standard laboratory tests and ultrasound, these parameters could help clinicians in evaluation and decision-making in their therapeutic approach for suspected AA. However, so far these cytokines have not been sufficiently examined in preoperative and postoperative laboratory monitoring of AA.

Confirmatory studies with a larger number of patients are required to prove reliability of these biomarkers in diagnosis and follow up of AA in children.

## Data Availability

The datasets used and/or analysed during the current study are available from the corresponding author on reasonable request.
